# Influence of dietary state and insulin on myocardial, skeletal muscle and brain [^18^F]-fluorodeoxyglucose kinetics in mice

**DOI:** 10.1186/2191-219X-1-8

**Published:** 2011-07-06

**Authors:** Michael C Kreissl, David B Stout, Koon-Pong Wong, Hsiao-Ming Wu, Evren Caglayan, Waldemar Ladno, Xiaoli Zhang, John O Prior, Christoph Reiners, Sung-Cheng Huang, Heinrich R Schelbert

**Affiliations:** 1 Department of Molecular and Medical Pharmacology, David Geffen School of Medicine at UCLA, Los Angeles, CA, USA; 2 Klinik und Poliklinik für Nuklearmedizin, Universitätsklinikum Würzburg, Würzburg, Germany; 3 The Crump Institute for Molecular Imaging, David Geffen School of Medicine at UCLA, Los Angeles, CA, USA; 4 Uniklinik Köln - Herzzentrum, Klinik III für Innere Medizin, Cologne, Germany; 5 Nuclear Medicine Division, Centre Hospitalier Universitaire Vaudois (CHUV University Hospital) and University of Lausanne, Lausanne, Switzerland

## Abstract

**Background:**

We evaluated the effect of insulin stimulation and dietary changes on myocardial, skeletal muscle and brain [^18^F]-fluorodeoxyglucose (FDG) kinetics and uptake *in vivo *in intact mice.

**Methods:**

Mice were anesthetized with isoflurane and imaged under different conditions: non-fasted (*n *= 7; "*controls*"), non-fasted with insulin (2 IU/kg body weight) injected subcutaneously immediately prior to FDG (*n *= 6), fasted (*n *= 5), and fasted with insulin injection (*n *= 5). A 60-min small-animal PET with serial blood sampling and kinetic modeling was performed.

**Results:**

We found comparable FDG standardized uptake values (SUVs) in myocardium in the non-fasted controls and non-fasted-insulin injected group (SUV 45-60 min, 9.58 ± 1.62 vs. 9.98 ± 2.44; *p *= 0.74), a lower myocardial SUV was noted in the fasted group (3.48 ± 1.73; *p *< 0.001). In contrast, the FDG uptake rate constant (*K*_i_) for myocardium increased significantly by 47% in non-fasted mice by insulin (13.4 ± 3.9 ml/min/100 g vs. 19.8 ± 3.3 ml/min/100 g; *p *= 0.030); in fasted mice, a lower myocardial *K*_i _as compared to controls was observed (3.3 ± 1.9 ml/min/100 g; *p *< 0.001). Skeletal muscle SUVs and *K*_i _values were increased by insulin independent of dietary state, whereas in the brain, those parameters were not influenced by fasting or administration of insulin. Fasting led to a reduction in glucose metabolic rate in the myocardium (19.41 ± 5.39 vs. 3.26 ± 1.97 mg/min/100 g; *p *< 0.001), the skeletal muscle (1.06 ± 0.34 vs. 0.34 ± 0.08 mg/min/100 g; *p *= 0.001) but not the brain (3.21 ± 0.53 vs. 2.85 ± 0.25 mg/min/100 g; *p *= 0.19).

**Conclusions:**

Changes in organ SUVs, uptake rate constants and metabolic rates induced by fasting and insulin administration as observed in intact mice by small-animal PET imaging are consistent with those observed in isolated heart/muscle preparations and, more importantly, *in vivo *studies in larger animals and in humans. When assessing the effect of insulin on the myocardial glucose metabolism of non-fasted mice, it is not sufficient to just calculate the SUV - dynamic imaging with kinetic modeling is necessary.

## Background

The development of high-spatial-resolution small-animal PET has opened a new field for translational research. With these dedicated devices, regional organ tissue radiotracer concentrations can be visualized and measured. Moreover, radiotracer tissue kinetic models initially established and validated in larger animals and in humans for measurements of regional functional processes can now be applied to small animals. It is thus possible to study myocardial substrate metabolism and its determinants in intact animals rather than in isolated hearts. Importantly, because PET allows simultaneous measurements of radiotracer uptake and tissue kinetics in multiple organs such as skeletal muscle, brain, and myocardium, system-wide response of individual organ metabolic rates to physiological or pharmacological stimuli can be evaluated.

The small organ size in these animals, together with limitations in blood sampling, poses considerable methodological challenges. Accordingly, only few investigations have attempted to measure glucose metabolic rates in the myocardium, skeletal muscle, and brain in mice or rats [1-6]; many of them addressed mainly methodological aspects. Earlier studies from our laboratory have already demonstrated the feasibility of determining the radiotracer arterial input function and the tissue kinetics in myocardium, skeletal muscle, and brain in intact mice [7-10]. The purpose of the current study was to determine, if myocardial [^18^F]-fluorodeoxyglucose (FDG) kinetics in mice in a non-fasting condition or a fasting condition differ after injection of insulin and furthermore assess the effect on FDG kinetics in the muscle and brain. Knowledge of the extent of these changes will assist in planning future experiments for assessing glucose metabolism to help decide if kinetic modeling is necessary or which metabolic state would be the most suitable to answer the scientific question.

## Methods

### Study design

Twenty-three male C57BL/6 mice (age 12-24 weeks, Charles River Laboratories Inc., Wilmington, MA, USA) were assigned to four study groups (Table 1). Non-fasted, fed *ad libitum *mice served as "control group" (*n *= 7). In the second group, defined as "non-fasted insulin" (*n *= 6), mice were injected subcutaneously with short-acting insulin (2 IU/kg body weight; Novolin R Human, Novo Nordisk Pharmaceutical Industries Inc., Clayton, NC, USA) 30-60 s prior to the intravenous (i.v.) FDG administration. In the third group, defined as "fasted, no insulin" (*n *= 5), mice were kept without chow overnight to assess the effects of fasting. Finally, in the fourth group, defined as "fasted, insulin" (*n *= 5), the effect of acute insulin administration immediately prior to the i.v. FDG on the FDG tissue kinetics was examined.

**Table 1:** Characteristics of the four study groups.

**Group no**.	*n*	Metabolic condition	Weight (g)
1	7	Non-fasted, no insulin; "controls"	28.9 ± 4.7
2	6	Non-fasted, insulin	28.3 ± 3.6
3	5	Fasted, no insulin	29.1 ± 4.1
4	5	Fasted, insulin	29.4 ± 5.1

All animals were kept on a normal 12-h day/night cycle, had free access to water and were studied between 8 and 10 am to minimize circadian variations of substrate metabolism. Standard chow (Teklad S-2335 Mouse Breeder Diet 7904, Harlan Teklad Animal Diets & Bedding, Indianapolis, IN, USA; 17.0% protein, 11.0% fat, and <3.5% fiber) was used. The study was approved by the UCLA Animal Research Committee and performed in accordance with NIH Guidelines for the Care and Use of Laboratory Animals.

### Animal preparation and imaging procedure

Mice were anesthetized by inhalation of 2% isoflurane (Isoflo, Abbott Laboratories, North Chicago, IL, USA) in 100% oxygen in an induction box heated to 36°C. The animals were placed on a heated PET-CT animal holder, which provided anaesthesia through a nose cone [11]. A 29 G needle, attached to a 5-7 cm long polyethylene catheter (PE 20; Intramedic, Clay Adams, Sparks, MD, USA) was inserted into the proximal tail vein.

A 60-minute microPET list mode data acquisition was started 2 - 5 seconds prior to an i.v. FDG bolus (18.1 ± 5.5 MBq in 30 μl). Five to 13 serial venous blood samples (warmed tail tip, 4-17 μl/sample) were collected during the study from the tail tip for determination of plasma FDG concentrations. Plasma glucose levels were measured before and following insulin administration (5-13 samples) using tail vein blood samples (~0.3 μl each), with glucose test strips (Therasense^® ^Freestyle^®^, Therasense Inc., Alameda, CA, USA). Blood loss due to blood sampling averaged 134.4 ± 40.0 μl, which was less than 10% of the total blood volume of a mouse.

A microCT study (microCAT™ II, Siemens Preclinical Solutions, Knoxville, TN, USA) was performed upon completion of the PET study.

### Image reconstruction and analysis

Small-animal PET was performed on a microPET^® ^Focus 220 system (Siemens Preclinical Solutions). Starting at the time of injection, the acquired list mode data were binned into 30 image frames (15 × 0.5, 1 × 2, 1 × 4, 1 × 6, 1 × 15, 3 × 30, 1 × 60, 1 × 120, 3 × 180, 3 × 900 s). Reconstruction incorporated a filtered backprojection algorithm with a ramp filter and a cutoff frequency of 0.5 of the Nyquist frequency to obtain an image pixel size of 0.4 × 0.4 × 0.8 mm and an inter-plane spacing and slice thickness of 0.8 mm in a 128 × 128 matrix. The image reconstruction software provided for correction of radioactivity decay, random coincidences, dead-time losses, and photon attenuation (microPET^® ^Manager v. 2.1.5.0; Siemens Preclinical Solutions). Photon attenuation was corrected for by CT-derived attenuation maps as described previously [12].

### Quantitative image analysis

The software AMIDE [13] was used for image display and volume of interest (VOI) analysis. A large cylindrical VOI was assigned to the whole body of the mouse (109 cm^3^), an ellipsoidal VOI to the brain (57.5 mm^3^) and four small, same-size box VOIs (2.2 mm^3 ^each) to the myocardium as visualized on the late phase PET images. Another small box shaped VOI (1.4 mm^3^) was assigned to a proximal foreleg muscle on the coregistered CT images. Finally, a cylindrical VOI (2.6 mm^3^) was placed into the left ventricle (LV) blood pool on the radiotracer first pass (early time frame) images.

Standardized uptake value (SUV) was calculated to normalize the radiotracer tissue concentrations to the injected dose and body weight according to the following equation:(1)

The injected dose was estimated from the total counts in the whole-body VOI assigned to the last image frame as described previously [14]. The tissue activity concentrations were obtained from the VOIs on the last 900-s image.

Radiotracer concentrations in myocardium were determined from the average counts in the four myocardial VOIs. For measurement of the radiotracer input function, blood sample radioactivity concentrations were determined in a high-energy γ counter (Packard Cobra II Auto Gamma, Perkin Elmer Inc., Wellesley, MA, USA). The input function was determined by a previously published method that uses the early portion (*t *≤ 1 min) of LV time-activity curve derived from image data, adjusted for delay, dispersion, and partial-volume effects, and two arterialized blood samples taken from the tail vein at about 45 and 60 min p.i. [8].

Time-dependent changes in the distribution of FDG concentrations in whole blood and plasma were corrected for by the following equation established previously by our group [15]:(2)

where cf is the correction factor, *t *the time in minutes after the FDG injection.

From the input function and the image-derived organ FDG concentrations, the FDG uptake rate constant (*K*_i_) was estimated with the Gjedde-Patlak graphical analysis [16,17](3)

whereas *C*_*T*_(*T*) and *C*_*p*_(*T*) are the tissue and plasma radioactivity concentrations at each sample time point *T *(4 to 22 min; [9]), *t *is the integration variable, and INT is the y-intercept of the graphical plot. All calculations were performed with the internet-based software "Kinetic Imaging System" [18]. Linearity of the graphical plot was confirmed visually. For all assessed organs a specific density of 1.00 g/ml was assumed.

Glucose metabolic rates (MR_gluc_) were estimated by MR_gluc _= (*K*_i _× *C*_gluc_)/LC, where *C*_gluc _is the glucose concentration in plasma and LC the lumped constant. A value of 1 was assumed for the lumped constant. Because of marked changes in plasma glucose levels after insulin administration, glucose metabolic rates were estimated only for the groups without insulin injection.

To evaluate group differences in the FDG plasma clearance, the input function was normalized for body weight and injected dose in the same way as the tissue data and expressed as SUV. Since the time points of blood sampling and plasma glucose measurements varied slightly from mouse to mouse, interpolation was applied for the inter-group analysis at predefined time points.

### Statistical analysis

Data are given with mean values and standard deviation. Differences in SUVs, uptake rate constants, metabolic rates, input functions, and plasma glucose levels in the non-fasted control animals compared to the other animal groups were evaluated for statistical significance by one-way ANOVA analysis. Intra-group differences in plasma FDG and glucose levels were evaluated using the Student's *t *test. When comparing more than two groups Bonferroni post-hoc corrections were applied. *p *values < 0.05 were considered to indicate statistical significance.

## Results

### Influence of fasting and insulin administration on plasma glucose and [^18^F]-activity concentrations

**Figure 1:**
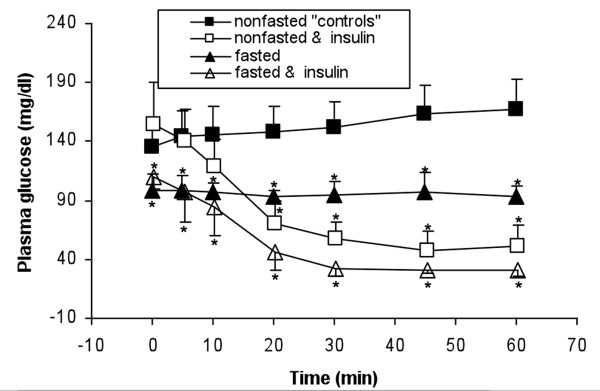
**Plasma glucose levels in the four groups of mice. Insulin injection resulted in a rapid decline of plasma glucose levels**. The insulin injected groups were shifted by 0.3 min to reduce overlay of error bars. * *p *< 0.05 vs. non-fasted controls by ANOVA and after Bonferroni correction.

Plasma glucose levels in the control group were significantly higher as compared to the fasted group (137 ± 17 *vs*. 98 ± 14 mg/dl; *p *= 0.009). Plasma glucose levels progressively increased in control animals within 60 min (166 ± 25 mg/dl; *p *= 0.003), but remained relatively constant in fasted animals (Figure 1). In both insulin groups, plasma glucose levels steeply declined; by 30 min, they had decreased to 63.0 ± 4.8% and 70.8 ± 7.3% of the initial values.

**Figure 2:**
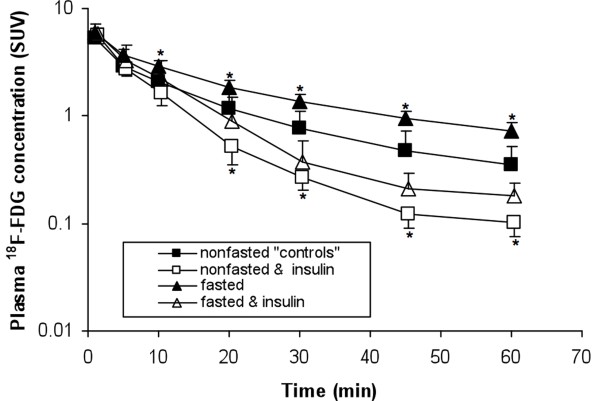
**Plasma FDG concentrations in the four groups of mice**. In both insulin groups FDG cleared from plasma more rapidly than in the groups without insulin injection. Y axis is in logarithmic scale. The insulin injected groups were shifted by 0.3 min to reduce overlay of error bars. * *p *< 0.05 *vs*. controls by ANOVA and after Bonferroni correction.

In fasted animals, plasma [^18^F]-activities declined less rapidly during the microPET study as compared to non-fasted controls (Figure 2). As early as 10 min p.i., [^18^F]-activity concentrations were higher in fasted animals (*p *= 0.048), suggesting that circulating FDG remained available longer for uptake into tissue.

Insulin administration was associated with a faster decline of [^18^F]-plasma activities especially in non-fasted controls. Significantly lower values were noted already after 15 min in the non-fasted insulin group and after 30 min in the fasted insulin group (Figure 2), consistent with insulin-stimulated higher whole-body glucose and FDG disposal rates.

### FDG uptake, uptake rate constants, and glucose utilization rates

**Figure 3:**
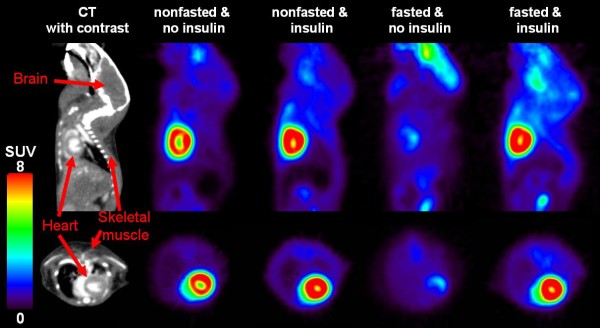
**Representative small-animal PET images**. Myocardial uptake is very similar in control animals as compared to the insulin-injected animals, but in the skeletal muscle, more FDG uptake can be noted after insulin injection. In fasted animals, myocardial FDG is markedly diminished. Sagittal (top) and transverse (bottom) views of one mouse of each group obtained 45-60 min p.i.

**Figure 4:**
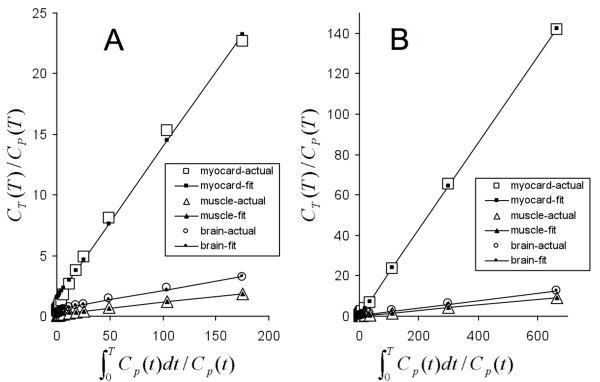
**Results of the graphical analysis**. Graphical analysis plots for myocardium, skeletal muscle, and brain in a non-fasted (A) and a non-fasted, insulin injected (B) mouse. The actual observed data points are compared to the least square regression line.

Figure 3 depicts representative PET images of the study groups. Compared to the non-fasted control group, SUV in myocardium of fasted mice was significantly lower (3.48 ± 1.73 *vs*. 9.58 ± 2.44; *p *< 0.001, Table 2). In skeletal muscle, a trend for a lower SUV was observed (0.40 ± 0.09 *vs*. 0.55 ± 0.11; *p *= 0.079). The brain SUV was found to be higher in fasted compared to control mice (2.87 ± 0.50 *vs*. 1.45 ± 0.42; *p *< 0.001). Interestingly, myocardial SUV in non-fasted control mice did not increase with insulin administration. Graphical analysis was applied in all mice to calculate tissue specific *K*_i_; correlation coefficients of least square fits averaged *R*^2 ^= 0.984 ± 0.007. Examples of Patlak plots of a non-fasted and an insulin treated mouse are shown in Figure 4.

**Table 2:** Organ standardized uptake values (SUV), FDG uptake rate constants (*K*_i_) and glucose metabolic rates (MR_gluc_)

	Myocardium	Muscle	Brain
SUV (45-60 min p.i.)			
Non-fasted	9.5 ± 2.4	0.55 ± 0.11	1.45 ± 0.41
Non-fasted and insulin	9.98 ± 1.06	0.97 ± 0.29*	0.96 ± 0.11*
Fasted	3.48 ± 1.73*	0.40 ± 0.09	2.87 ± 0.50*
Fasted and insulin	9.35 ± 1.62	1.00 ± 0.24*	1.73 ± 0.41
K_i _(ml/min/100 g)			
Non-fasted	13.44 ± 3.93	0.73 ± 0.25	2.24 ± 0.53
Non-fasted and insulin	19.79 ± 3.34*	1.89 ± 0.86*	2.52 ± 0.58
Fasted	3.34 ± 1.92*	0.36 ± 0.11*	2.99 ± 0.39
Fasted and insulin	20.01 ± 12.70	2.04 ± 1.80*	3.56 ± 1.21
MR_gluc _(mg/min/100 g)			
Non-fasted	19.41 ± 5.39	1.06 ± 0.34	3.21 ± 0.53
Fasted	3.26 ± 1.97*	0.34 ± 0.08*	2.85 ± 0.23

*p < 0.05 *vs*. non-fasted controls by ANOVA and after Bonferroni correction.

In parallel to calculated SUVs, myocardial K_i _values in the fasted group were found to be significantly lower (-75.5%) compared to non-fasted controls (Table 2; *p *< 0.001). Insulin administration produced a significant increase in *K*_i _values in both groups. In contrast to SUV, insulin led to an increase in *K*_i _in non-fasted animals, on average 43.5% (*p *= 0.030) compared to controls. In fasted mice, insulin produced myocardial *K*_i _values, which were more than 300% higher than without (20.0 ± 12.7 *vs*. 3.3 ± 1.9; *p *< 0.001). Insulin stimulation led to similar myocardial *K*_i _values in fasted mice as in non-fasted controls despite a significant difference in plasma glucose levels before the PET study.

In skeletal muscle, concordant changes in *K*_i _were noted with the highest values after insulin administration (Table 2). Brain *K*_i _values were not affected by insulin or fasting.

Consistent with the *K*_i _changes, MR_gluc _considerably differed between the non-fasted and the fasted animals in the insulin-sensitive organs (Table 2). Fasting was associated with an 81% reduction in myocardial and a 68% reduction in skeletal muscle MR_gluc _when compared to non-fasted controls.

## Discussion

In this study, we investigated the effect of the metabolic condition on the biodistribution and uptake rates of FDG in mice. We found that FDG plasma clearance rates depend on the dietary state and on insulin stimulation. It was lowest in fasted animals, probably reflecting a diminished whole-body glucose disposal rate, as reflected in the lower *K*_i _for myocardium and skeletal muscle and possibly related to inhibitory effects of high plasma free fatty acid concentrations on tissue uptake and a low membranous expression of GLUT4. The marked increase in plasma FDG clearance after insulin administration corresponded to an increase in *K*_i _for myocardium and skeletal muscle. Because skeletal muscle constitutes a significant fraction of the body mass in mice [19], the observed insulin-induced increase in plasma clearance rates can be attributed to an increase in skeletal muscle FDG uptake and, thus, an increase in whole-body glucose disposal rates.

Importantly, graphical analysis could be performed successfully (Figure 4), even though plasma glucose concentrations differed between groups and markedly changed over time. The finding suggests that FDG transmembranous transport and phosphorylation rates remained constant throughout the study, despite significant changes in blood glucose levels. Insulin prompted marked increases in transmembranous glucose transport and phosphorylation rates, as reflected by *K*_i_. In addition, progressively declining plasma glucose reduced substrate competition for FDG transport and phosphorylation, resulting in an increased FDG uptake rate constant that probably compensated for any FDG clearance in tissue, thus creating the apparent irreversible uptake of FDG (i.e., linearity on the Patlak plot). In contrast, insulin had no effect on cerebral *K*_i_, most likely due to the absence of GLUT4 in the brain and insulin-independent cerebral glucose metabolic rates.

Changes in myocardial SUV due to fasting and insulin for the most part corresponded to changes in *K*_i_. However, this does not hold true for the non-fasted controls and the non-fasted insulin group; here, myocardial *K*_i _was found to be increased after insulin injection even though myocardial SUVs were similar in both groups. This disparity may be related to a shortcoming of the SUV as a widely employed measure of tissue FDG uptake. Inherent in the use of SUV is the assumption of a constant radiotracer input function. However, radiotracer input functions markedly differed between study groups. In the insulin-treated animals, FDG cleared more rapidly from plasma so that a decrease in circulating radiotracer activities was associated with a disproportionately lower myocardial SUV.

Brain MR_gluc_, estimated only in the non-insulin-treated mice with relatively stable plasma glucose levels averaged in a non-fasted state about 3.2 mg/min/100 g, a value very comparable to that reported by our group in mice with arterial catheters (2.2 mg/min/100 g) [9]. In the skeletal muscle, the MR_gluc _in non-fasted control mice (about 1.1 mg/min/100 g) again was of a similar order of magnitude as those reported for humans during insulin clamping (about 4.9 mg/min/100 g) [20]. In the myocardium, the MR_gluc _in mice (19.4 and 3.3 mg/min/100 g in non-fasted and fasted mice, respectively) again were similar to those in humans (12.4 and 4.3 mg/min/100 g after glucose loading and fasting, respectively) [21].

Reduced heart and skeletal muscle glucose or FDG metabolism under insulin clamping conditions in patients with type 2 diabetes and coronary artery disease (CAD) has been reported [22]. It has also been reported that there was no difference in myocardial glucose metabolism under glucose loading and under insulin clamping in patients with CAD [23]. Reduced myocardial FDG metabolism under fasting, glucose loading, and insulin clamping in patients with type 2 diabetes without CAD has been reported [24]. On the other hand, myocardial glucose metabolism in response to insulin clamping is not always parallel to that in skeletal muscle and/or whole-body glucose metabolism. For instance, myocardial glucose metabolism was increased or unchanged with insulin clamping in patients with essential hypertension, although skeletal muscle and whole-body glucose metabolism were significantly reduced with insulin clamping [25]. Myocardial glucose metabolism was not reduced in patients with type 2 diabetes and essential hypertension, even though skeletal muscle and whole-body glucose metabolism was reduced [26]. In patients with hypertriglyceridemia without hypertension and diabetes, myocardial glucose metabolism was not significantly reduced under insulin clamping, but skeletal muscle and whole-body glucose metabolism was significantly reduced [27]. These clinical results and the current results, which showed different responses to insulin stimulation regarding glucose metabolism between heart and skeletal muscle, indicate that myocardium and skeletal muscle might have different mechanisms for regulation of glucose or FDG uptake in response to insulin-stimulation or insulin clamping.

Regardless of absolute values of *K*_i _and MR_gluc_, it is important to note that dietary changes as well as insulin administrations exerted responses in mice that are comparable to those in humans. Fasting diminished the whole-body glucose disposal rates and glucose uptake in myocardium and skeletal muscle. Conversely, insulin raised whole-body FDG and glucose disposal rates and increased transmembranous transport of FDG and, by inference, glucose into myocardium and skeletal muscle.

**Figure 5:**
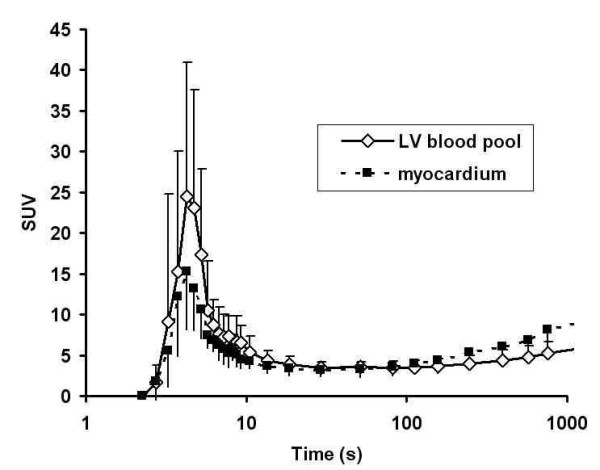
**Distribution of FDG in the LV blood pool and the myocardium in the early phase of the study**. Initial phase of the radiotracer input function in a "control animals" from VOIs assigned to LV blood pool and myocardium. The time axis is plotted logarithmically. A spillover of radioactivity could be observed no earlier than 100 s p.i.. Since only the first 60 s of the LV blood pool time-activity curve were used for the image-derived input function the influence activity spillover from the myocardium into the blood pool can be expected to be negligible.

Some limitations have to be considered when interpreting our findings. Firstly, no corrections were made for spillover of activity between arterial blood and myocardium. Activity spillover from myocardium into the LV blood pool VOI during the initial bolus passage and the first 60 s used for determining the input function was likely to be low as seen on the first-pass time-activity curves (Figure 5). Blood sampling for determination of FDG plasma concentrations in the current study eliminated spillover effects on the late phase of the arterial input function; the validity of this method has been shown before [8]. Secondly, it has been reported in humans, that the lumped constant in the myocardium is influenced by the insulin levels [28]. However in the current study, a fixed value of 1.0 was used for all groups because insulin levels were not available and also because lumped constants have yet to be determined. Thirdly, isoflurane anesthesia is known to affect myocardial glucose uptake [29,30] and may have influenced the results. These limitations are, however, unlikely to reduce the validity of the inter-group comparison, because the animals in the four study groups were of similar body weight and, by inference, had similarly sized organs and were exposed to the same anesthesia. Identification of effects of substrate competition on organ glucose utilization rates would have been useful but would have required measurements of plasma free fatty acid and lactate levels. The blood volume of mice limits the amount of blood that can be taken from the animals. Blood sampling was minimized to avoid excessive stress and its effect on the metabolic state, as reported previously [31]. The use of arterialized venous blood samples for estimating glucose metabolic rates has been well established in humans [32,33], as well as in rats and mice [1,8].

## Conclusions

In this study, we not only measured organ SUVs, uptake rate constants, and glucose metabolic rates in intact mice; we were also able to monitor alterations induced by dietary changes and insulin administration. When assessing the effect of insulin on the myocardial glucose metabolism of non-fasted mice, it is not sufficient to just calculate the SUV; dynamic imaging with kinetic modeling is necessary. The observed dietary and insulin-induced changes in organ metabolic rates, as observed in the current study, are similar to those reported for humans.

## Competing interests

The authors declare that they have no competing interests.

## Authors' contributions

MCK performed all animal experiments, developed the methodology, analyzed the data, and wrote the manuscript. DBS provided advice in the conception of the study in terms of methodology (heated animal chamber, image reconstruction) and critically reviewed the manuscript. KPW helped in the kinetic analyses and critically reviewed the manuscript. HMW provided advice in the conception of the study and interpretation of the data, performed experiments, and critically reviewed the manuscript. EC performed the blood sampling and analyzed the data as well as critically reviewing the manuscript. WL assisted in conducting the animal studies, gave valuable input on animal handling, performed the image reconstructions, and reviewed the manuscript. XZ helped perform the animal studies, gave valuable input on the biological aspects and reviewed the paper. JOP helped to statistically analyze and interpret the data and considerably improved the manuscript in writing. CR provided intellectual input, help in the statistics, and reviewed the manuscript. SCH is the co-PI of this study and involved in the design and interpretation of the kinetic modeling as well in writing the manuscript. HRS is the PI of the study and is involved in all aspects of this work from design to writing. All authors read and approved the final manuscript.
